# Whole Blood Transcriptome Analysis in Dairy Ewes Fed a Dietary Grape Pomace Supplementation

**DOI:** 10.3390/vetsci11110536

**Published:** 2024-11-01

**Authors:** Andrea Ianni, Francesca Bennato, Camillo Martino, Maria Antonietta Saletti, Francesco Pomilio, Giuseppe Martino

**Affiliations:** 1 Department of BioScience and Technology for Food, Agriculture, and Environment, University of Teramo, 64100 Teramo, TE, Italy; aianni@unite.it (A.I.); fbennato@unite.it (F.B.); 2 Department of Veterinary Medicine, University of Perugia, 06126 Perugia, PG, Italy; camillo.martino@studenti.unipg.it; 3 Food Hygiene Unit, NRL for L. Monocytogenes, Istituto Zooprofilattico Sperimentale dell’Abruzzo e del Molise “G. Caporale”, 64100 Teramo, TE, Italy; m.saletti@izs.it (M.A.S.); f.pomilio@izs.it (F.P.)

**Keywords:** dairy ewes, animal welfare, grape pomace, transcriptomics, RNA-seq

## Abstract

The present study aims to evaluate the effect of a dietary supplementation with 10% grape pomace (GP) on the whole blood transcriptome of lactating ewes. By applying a log_2_FC higher than 0.5 or lower than −0.5 and a false discovery rate (FDR) <0.05, the down-regulation of genes coding for plexin C1, ethanolamine kinase 1, tax1-binding protein 1, transmembrane 9 superfamily member 2, and Beclin-1 was observed in animals that received the dietary supplementation. This aspect was also accompanied by a reduction in the blood activity of matrix metalloproteinase 9 (MMP-9; *p* < 0.05), a gelatinase commonly involved in both acute and chronic pathological events. The ELISA test on other factors involved in inflammatory processes, interleukin 1 (IL-1) and tumor necrosis factor α (TNF-α), as well as in the antioxidant response, glutathione peroxidase (GPx), and catalase (CAT), did not reveal any significant changes (*p* > 0.05). Overall, the introduction of GP in the diet of ewes gave indications of greater efficacy in preserving animal welfare, with interesting cues regarding the valorization of a by-product with a high biological value.

## Simple Summary

The grape handling required for wine production is responsible for the accumulation of significant quantities of solid organic waste represented by the grape pomace. The use of this matrix as an ingredient for the diet of lactating ewes has proved to be an effective strategy in preserving animal welfare, with interesting insights into the down-regulation of factors involved in inflammatory processes. The obtained results confirm the sustainability of this strategy, with the potential for implementation in the circular economy.

## 1. Introduction

For the year 2022, International Organization of Vine and Wine (OIV) reported a global grape production of about 77 million tons, with the wine industry being responsible for the use of about half of the entire grape production. Grape pomace (GP) represents the main solid by-product resulting from the production processes implemented by the oenological industry, and is characterized by the presence of interesting bioactive compounds which are associated with several biochemical activities, especially polyphenols (2–6.5%), such as flavonoids, phenolic acids, tannins, and proanthocyanidins [1]. The use of by-products of the agro-food industry as matrices of interest for zootechnical feeding represents a topic for which considerable interest has developed in recent years [2]. This strategy, which can be easily framed within the circular economy, provides for the recovery and valorization of organic matrices that would otherwise have to be managed as waste, with several environmental and economic issues related to their disposal [3]. With reference to the livestock sector, one of the matrices on which much attention has been paid is grape pomace (GP), which mainly comes from the grapes processed for wine production [4]. This by-product, in the same way as other waste matrices of vegetal origin, represents a rich source of potent bioactive compounds belonging to the large family of polyphenols, which could display a wide range of beneficial effects in several biological processes [5]. Furthermore, from a nutritional point of view, GP is even characterized by the presence of fibers and significant concentrations of polyunsaturated fatty acids (PUFAs), especially linoleic acid, and fibers, which give an additional health value to this natural matrix [6,7].

Several studies conducted in recent years addressed the use of GP as a supplement in the diets of dairy ruminants, with a particular focus on the effects induced on the quality of milk and derived dairy products. Overall, supplementation of ruminants’ diets with GP seems to induce a general modification of the fatty acid composition in both milk and cheeses, with an interesting reduction in the SFA/UFA associated with an increased amount of PUFAs and a parallel decrease in SFA content [8]. This condition is very important for consumers, since it is generally associated with advantages at the level of the cardiovascular system, due to the fact that PUFAs are considered capable of decreasing the amount of low-density lipoprotein cholesterol and serum cholesterol, while SFAs correlate with high levels of serum cholesterol [9]. In addition to this, evaluations carried out on dairy products subjected to ripening have highlighted a greater resistance to the oxidative processes, a phenomenon that was attributed to the presence in these matrices of compounds with antioxidant activity, presumably of phenolic nature, directly derived from the GP ingested by the animals [10].

In addition to the aspects closely associated with the quality of products of animal origin, some studies also focused their attention on the effect induced by GP intake on animal welfare, starting from the assumption that the consumption of ingredients rich in bioactive compounds could better preserve animal health and, hopefully, improve their production performances [11]. One of the most reliable tools used to investigate this aspect is represented by whole blood transcriptome profiling, which allows the set of transcripts present at the level of the blood lymphocytes to be sequenced, providing information on any changes in gene expression induced by diet.

In a trial involving Friesian calves fed a dietary GP supplementation, whole blood RNA sequencing was effective in highlighting positive effects in the modulation of the pathway responsible for cholesterol biosynthesis with the consequent reduction in circulating cholesterol [12]; the RNA-Seq data specifically showed the down-regulation of five genes involved in the cholesterol biosynthesis pathway (FDFT1, MSMO1, NSDHL, SC5D, and SQLE). A study conducted by Pauletto et al. [13] highlighted that the feeding of dairy cows with GP was able to down-regulate some genes involved in the processes of oxidative stress and inflammation. Specifically, a reduction in the expression of the DNAJA1 gene was highlighted. This gene is also known as heat shock protein (HSP 40) and plays a key role in physiological and stress conditions, and in the processes of stabilization and correct folding of nascent proteins. In lactating dairy ewes, the ability of dietary grape seed to positively modulate the immune function has already been described [14]. In lambs, the GP intake significantly increased the activity of reduced glutathione and catalase (CAT) in blood and tissues, contributing to the reduction in oxidative damage in lipids and proteins [15]. Additionally, in vitro studies performed using crude GP extract highlighted a significant modulatory effect on the activity of matrix metalloproteinases (MMPs), essential factors involved in the inflammatory process [16].

In the past, we have investigated the transcriptomic signature of Friesian calves fed a diet enriched with GP [12], and, more recently, we reported the nutrigenomic effect induced by long-term dietary supplementation in dairy cows [13]. Considering the hypothesis that dietary GP inclusion, presumably due to the typology of phenolic compounds that characterize the matrix, is able to modulate the gene expression of ruminants, we characterized the whole-transcriptome profile in lactating dairy sheep and investigated the effects on factors involved in the animal response to oxidative and inflammatory stress.

## 2. Materials and Methods

The experimentation was conducted in a commercial company where, in autumn, the diet of lactating dairy ewes is habitually integrated with GP derived from the vinification processes carried out in the months of August and September. This is the reason why no farming practices other than those normally adopted have been introduced. Additionally, the trial was planned according to the European regulation regarding the protection of animals used for scientific purposes: Directive 2010/63/EU of the European Parliament (European Union, 2010) and Directive 86/609/EEC (European Economic Community, 1986) [17,18]. Blood sampling was performed only at the end of the trial by authorized veterinarians, concurrent with planned blood withdrawal for the prophylaxis protocols; therefore, no ethical declarations were deemed necessary.

### 2.1. Experimental Design and Blood Sample Collection

This study is part of a project focused on the evaluation of the effects of a GP-supplemented diet on both the qualitative features of milk and its derivatives and the metabolic aspects of lactating Assaf ewes. For this reason, the experimental plan has been already reported in a previous paper [19]. Briefly, the study involved forty-six dairy ewes. All the animals were in the first part of the lactation (<45 days in milk) and, as a result, were homogeneous in terms of milk production (1.40 ± 0.20 kg/head/die), body weight (75 ± 5 kg), and number of lactations (third lactation). Animals were randomly and equally distributed into two groups: a control group (CG) that received a standard diet, and an experimental group (EG) fed a diet containing GP (10% on a dry matter (DM) basis) as a substitute for beet pulp. The GP used in the trial, derived from red grape (*Vitis vinifera* L.), was obtained through a process that involved the preliminary elimination of ethyl alcohol through a step of fermentation and subsequent treatment with steam. In a second phase, the by-product was treated with water at 90 °C in order to recover the tartaric acid, and only at this point was drying performed, followed by flour production. The animals were kept in two separated areas within a closed barn and fed individually during the milking. Specifically, each animal received, for 60 days, alfalfa hay ab libitum and, in correlation with the two daily milkings (8:00 and 18:00), a custom-formulated concentrate (1 kg/day per head) whose ingredients and chemical composition have already been described in the paper of Bennato et al. [19]. The beginning of the trial was preceded by a 10-day period of adaptation to the experimental diet, during which, for each animal, the concentrate was gradually increased up to 1 kg of DM. This was achieved on day 10, which was considered time zero. The end of the trial, which lasted about 70 days, was made to coincide with the blood sampling scheduled by the veterinarians of the national health system for normal prophylaxis measures. By taking advantage of this circumstance, it was possible to obtain individual samples of whole blood (WB) to be subjected to hematochemical analysis and RNA extraction for transcriptomic investigations. Specifically, at the end of the trial, the fasting blood samples were obtained before the morning milking from a total of 10 randomly selected animals: 5 from the CG and 5 from the EG. To obtain the blood serum to be subjected to biochemical evaluations, 2.5 mL of blood samples was kept at room temperature for a time interval between 30 and 45 min to favor clot formation; then, a centrifugation step was performed at 1000× *g* for 15 min at room temperature. The obtained supernatant was recovered, aliquoted, and stored at −20 °C until analysis. The same animals were also sampled for the RNA-Seq analysis, by drawing blood that was collected in 2.5 mL PAXgene™ tubes (Becton Dickinson Biosciences, Franklin Lakes, NJ, USA), stored overnight at room temperature, and then stored at −20 °C until RNA extraction, as prescribed by the manufacturer.

### 2.2. Blood Analysis

Ewes’ blood and serum samples were analyzed at the Veterinary and Public Health Institute of Teramo (Teramo, Italy) using a laser-based hematology analyzer with software applications for animal species (ADVIA 120 hematology system, Siemens, Munich, Germany), and following the routine procedure of the institute with regard to the instrument settings and data validation. Regarding the blood hematology, the following parameters were analyzed: white blood cell count (WBC), red blood cell count (RBC), hemoglobin (HGB), hematocrit (HCT), mean corpuscular volume (MCV), mean corpuscular hemoglobin (MCH), mean corpuscular hemoglobin concentration (MCHC), red cell distribution width (RDW), hemoglobin distribution width (HDW), platelet count (PLT), mean platelet volume (MPV), neutrophils, lymphocytes, monocytes, eosinophils, basophiles, and large uncolored cells (LUCs). The serum biochemistry was evaluated using an automatic analyzer (ILAB 650, Instrumentation Laboratory-Werfen, Milan, Italy) in order to evaluate the parameters GOT (AST), triglycerides, total proteins, amylase, gamma GT, GPT (ALT), albumin, azotemia (BUN), cholesterol, glucose, total bilirubin, creatinine, alkaline phosphatase (ALP), serum calcium, uric acid, and serum iron.

### 2.3. Library Preparation, RNA Sequencing, and Analysis

The RNA analysis, including the steps concerning the extraction and the bioinformatic evaluations, was conducted by an external company (Genomix4life SRL, Baronissi, Salerno, Italy). Total RNA was extracted using TRIzol and following the manufacturer’s instruction (Thermo Fisher Scientific, Waltham, MA, USA). The RNA amount obtained from each sample was determined with a Nanodrop One (Thermo Fisher Scientific, Waltham, MA, USA) and its purity was defined by exploiting the TapeStation 4200 (Agilent Technologies, Santa Clara, CA, USA). Indexed libraries were prepared from 500 ng/ea purified RNA with a TruSeq Stranded mRNA Sample Prep Kit (Illumina) according to the manufacturer’s instructions. The library quantification was performed by using the TapeStation 4200 in association with a Qubit fluorometer (Thermo Fisher Scientific, Waltham, MA, USA), then pooled such that each index-tagged sample was present in equimolar amounts, with the final concentration of the pooled samples equal to 2 nM. The pooled samples were subject to cluster generation and sequencing using an Illumina NextSeq 550 System (Illumina, San Diego, CA, USA) in a 2 × 75 paired-end format [12]. The raw sequences (fastq files) were subjected to a quality control based on the use of the FastQC tool (Version 0.11.8) for high-throughput sequence data [20], available on http://www.bioinformatics.babraham.ac.uk/projects/fastqc (accessed on 28 November 2023). The bioinformatic tool cutadapt (version 2.5) [21] was then used to allow the removal of the adapter sequences as well as the very short reads (reads length < 20). The mapping of paired-end reads was performed using STAR (version 2.7.5c) [22], with standard parameters, on reference genome assembly Ovis aries (sheep) obtained from NCBI (GCF_002742125.1_Oar_rambouillet_v1.0). For each sequenced sample, the expressed transcripts were quantified by exploiting the feature Count algorithm (version 2.0). An ad hoc R script using Bioconductor package DESeq2 was performed to normalize the data and to determine the differentially expressed genes [23]. A false discovery rate (FDR) less than or equal to 0.05 (FDR ≤ 0.05) was taken into account for the identification of differentially expressed genes (DEGs). A useful step in an RNA-Seq analysis is often the evaluation of the general similarity between the samples. To do this, we derived the heatmap sample-to-sample based on Euclidean distances and principal component analysis (PCA) among all samples in each condition considered.

### 2.4. Protein–Protein Interaction Analysis (STRING)

The proteins corresponding to the differentially expressed transcripts were considered in order to analyze the protein–protein interaction network through the STRING software (version 11.5; http://string-db.org/ (accessed on 6 December 2023)). The interaction score was set at 0.9, the highest permitted by the software, with the aim of avoiding false positives; furthermore, a 5% FDR (medium stringency) was selected. The output of this evaluation is represented by a figure with up to seven lines predicting different types of interaction.

### 2.5. Enzyme Linked Immunosorbent Assay of Serum Cytokines and Antioxidant Enzymes

Commercial kits (MyBioSource, Inc., San Diego, CA, USA) were used to determine the amount of interleukin 1 (IL-1; kit code: MBS034397) and tumor necrosis factor α (TNF-α; kit code: MBS2019710), and the activities of glutathione peroxidase (GPx; kit code: MBS841725) and catalase (CAT; kit code: MBS034397) in undiluted serum samples. The quantity of the cytokines TNF-α and IL-1 was determined with an ELISA kit based on antibody–antigen interactions and a horseradish peroxidase (HRP) colorimetric detection system to identify the antigen targets. The presence of IL-1 and TNF-α was detected at 450 nm and then expressed in pg/mL. GPx activity was spectrophotometrically determined at 412 nm by measuring the rate of formation of oxidized glutathione (GSSG); activity was expressed as nmol/min/mL. CAT activity was evaluated using a kit that exploits the peroxidase function of the enzyme. In the presence of an appropriate concentration of H_2_O_2_, CAT reacts with methanol to produce formaldehyde, which, in turn, is able to stimulate the chromogenic reaction. The absorbance of the reaction products was measured at 540 nm, and results expressed as nmol/min/mL. All these evaluations were performed through an ELISA microplate reader (EnSpire 2300 multireader; PerkinElmer, Waltham, MA, USA).

### 2.6. Gelatin-Zymography of Matrix Metalloproteinases 2 and 9

Gelatin-zymography has been used to characterize the gelatinolytic activity attributable to matrix metalloproteinases 2 (MMP-2) and 9 (MMP-9) in serum samples. For this purpose, volumes of each sample containing 5 µg of total proteins were diluted in a buffer without reducing conditions, and resolved by 8% SDS-PAGE containing type B gelatin (Sigma Aldrich, Milan, Italy) 0.15 mg·mL^−1^. The gels were then left in a renaturation buffer (50 mM Tris-HCl pH 8.0, containing 2.5% Triton X-100) for 45 min in order to remove the SDS. After incubation for 24 h in a developing buffer (50 mM Tris-HCl pH 8.0, containing 5 mM CaCl_2_, 200 mM NaCl and 0.02% Brij 35) responsible for the enzyme renaturation and activity recovery, gels were stained in a 0.1% solution of Coomassie blue R250 in 40% (*v*/*v*) methanol and 10% (*v*/*v*) acetic acid, and the visualized bands were analyzed for intensity by exploiting the software ImageLab 6.0.1.

### 2.7. Statistical Analysis

JMP Pro 14 software (SAS Institute Inc., Cary, NC, USA) was used for statistical analysis of blood samples (hematology parameters; MMP-2 and MMP-9 activities; IL-1 and TNF-α levels; GPx and CAT activities). As the ewes were fed individually, the animal was the experimental unit. Data were tested for normality and mean values belonging to the two experimental groups were compared using Student’s *t*-test. Differences associated with *p*-values lower than 0.05 and 0.01 were considered significant. The transcriptomic signature statistical analysis was performed with R (version 4.0.2), which was used to create a matrix of all genes expressed in all samples with the corresponding read-counts. The counts were specifically divided by sample-specific size factors determined by the median ratio of gene counts relative to geometric mean per gene, and the Wald test is commonly used for hypothesis testing when comparing two groups, as reported in the Deseq2 package [24].

## 3. Results

### 3.1. Hematology and Serum Biochemistry

In order to evaluate the general state of health of the lactating ewes at the end of the trial, blood analyses were first performed with the specific aim to evaluate the cellular whole blood count and characterize the main biochemical indicators. Table 1 reports the complete whole blood count evaluated in CG (n = 5) and EG (n = 5). None of the analyzed cell families showed significant differences as an effect of the dietary supplementation (*p* > 0.05).

Even for the serum biochemical parameters (Table 2), the dietary treatment was found to be unable of inducing a significant modification, although it should be highlighted that there was an increasing trend of the serum iron content in the animals that received the GP supplementation (130.00 µg/dL vs. 166.17 µg/dL in CG and EG respectively), with a *p*-value close to the significance level (0.07).

### 3.2. Influence of Dietary Grape Pomace Supplementation on Blood Transcriptome

**Figure 1:**
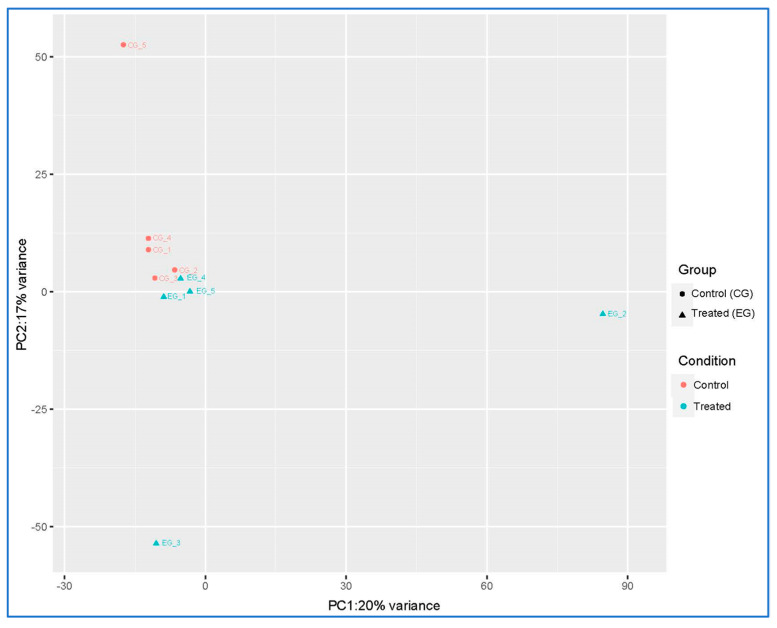
Principal component analysis (PCA) of genes expressed in whole blood samples obtained from ewes fed a standard diet (control group; CG) and the grape pomace dietary supplementation (experimental group; EG).

The transcriptomic signature of the animals involved in the trial was characterized by sequencing the RNA isolated from whole blood. In order to evaluate the general similarity between the samples, and therefore the separation existing between the two experimental groups, principal component analysis (PCA) was performed (Figure 1).

By combining an FDR lower than 0.05 with a log_2_FC higher than 0.5 or lower than −0.5, five DEGs were identified, all of which were down-regulated in lactating ewes that received the GP dietary supplementation (Table 3).

**Figure 2:**
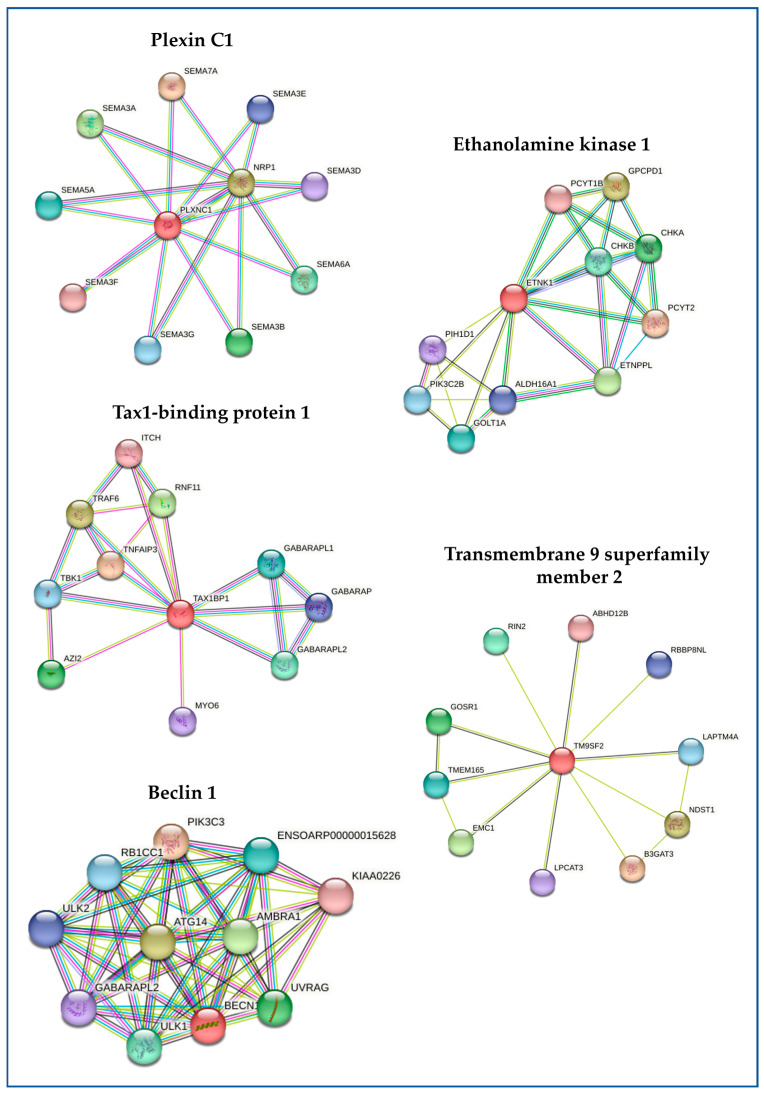
Interaction network of proteins corresponding to the differentially expressed genes (DEGs) identified through the blood transcriptome characterization. By making reference to the *Ovis aries* database, the software STRING 12.0 was effective in building the interaction network for each protein identified. Interactions are reported with different colors: cyan is from curated databases, magenta is experimentally determined, dark green is gene neighborhood, red is gene fusion, blue is gene co-occurrence, light green is text-mining, black is co-expression, and light blue is protein homology.

Based on the NCBI platform (ncbi.nlm.nih.gov/gene/?term (accessed on 6 December 2023)), the DEGs’ corresponding proteins were identified. The proteins involved are specifically Plexin C1, Ethanolamine kinase 1, Tax1-binding protein 1 isoform X1, Transmembrane 9 superfamily member 2, and Beclin-1. To identify molecular pathways associated with down-regulated sequences in EG samples, we interrogated STRING using the highest available interaction score (0.9) in order to increase stringency and avoid possible uncorrected data interpretation. The simultaneous insertion of the proteins corresponding to the five DEGs did not highlight reciprocal interactions from a functional point of view; however, the analysis was useful for identifying the factors most involved in the single interaction processes (Figure 2), and therefore provided a better understanding of the biochemical role of these proteins.

### 3.3. Analysis of Pro-Inflammatory Cytokines and Enzymes Involved in the Antioxidant Response

**Figure 3:**
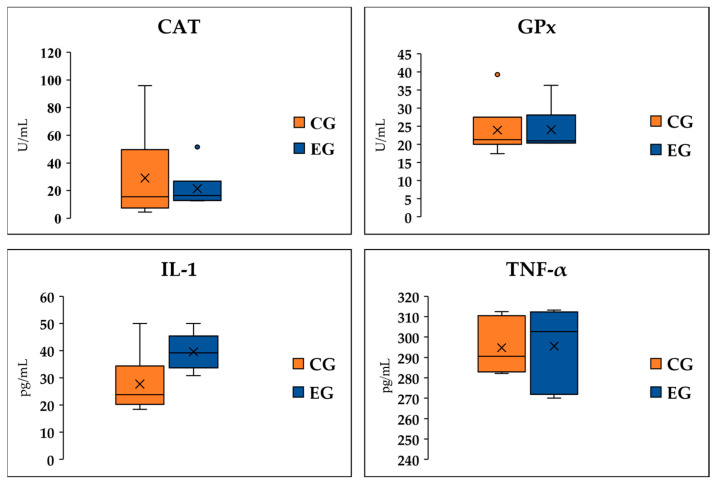
ELISA evaluation of the activity of two enzymes of the antioxidant response, glutathione peroxidase (GPx) and catalase (CAT), and the concentration of two pro-inflammatory cytokines, inteleukin-1 (IL-1) and tumor necrosis factor-α (TNF-α). The analysis was performed on blood serum samples obtained from ewes fed a standard diet (CG; n = 5) and the grape pomace dietary supplementation (EG; n = 5).

Reference to ELISA assays was made in order to evaluate the activity of two enzymes of the antioxidant response, GPx and CAT, and the concentration of two pro-inflammatory cytokines, IL-1 and TNF-α. In all monitored conditions (Figure 3), the dietary GP supplementation did not induce significant changes in blood serum (*p* > 0.05).

### 3.4. Evaluation of Inflammatory Mediators: The Gelatinases

**Figure 4:**
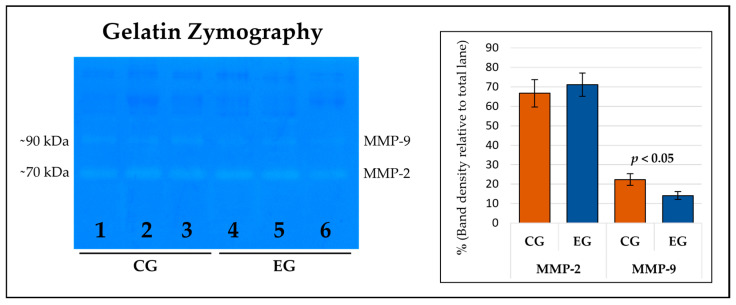
Representative zymography on serum blood samples obtained from ewes fed a standard diet (CG; lanes 1–3; n = 5) and the grape pomace dietary supplementation (EG; lanes 4–6; n = 5). In the histogram, the data are reported as a percentage of density of the band of interest, referring to the total calculated in the reference lane. The evaluation was effective in highlighting the activity of matrix metalloproteinases 2 (MMP-2) and 9 (MMP-9).

The zymographic approach was effective in highlighting the enzymatic activities associated with the two major gelatinases, MMP-2 (gelatinase-A) and MMP-9 (gelatinase-B), at the level of blood serum samples. As shown in Figure 4, MMP-2 (~70 kDa) did not undergo significant changes as a consequence of the dietary supplementation with GP, while in the case of MMP-9 (~90 kDa) it seems that the experimental diet was effective in inducing a significant reduction in the enzymatic activity (*p* < 0.05).

## 4. Discussion

In recent years, by-products from the agri-food chain have been used as dietary ingredients for animals of zootechnical interest. With specific regard to GP, the main solid by-product from the wine production process, numerous experiments have been carried out on ruminants, highlighting positive effects on the main qualitative parameters of both dairy and meat production, with a general increase in concentration of linoleic acid [8,9].

With specific regard to small ruminants, the influence induced by this kind of dietary approach on animal health and metabolism has still been little studied. In this study, we characterized the transcriptome in the peripheral blood of dairy ewes fed a GP supplementation. In previous studies performed on calves and dairy cows, this approach was found to be effective in highlighting DEGs with high accuracy and sensitivity [12,13]; furthermore, gene expression profiling in blood should be helpful in obtaining information potentially useful to reflect the molecular and biochemical mechanisms occurring in other tissues or organs [25].

An evaluation of the general health status of the animals involved in the experimentation was preliminarily conducted through the evaluation of blood cell count and the characterization of specific parameters in serum samples. None of the aspects just indicated showed significant differences, although it should be noted that for the iron content there is an increasing trend in the EG samples. Iron is a micronutrient that is well represented in grape [26] and, together with other trace elements, such as zinc or selenium, is important for the maintenance of normal metabolism and productivity in farm animals. Specifically in the presence of a bacterial infection, the blood levels of this element decrease as a non-specific host-defense mechanism, therefore representing an indicator of the natural immune function [26]. The observed result, therefore, represents a point of interest that could argue in favor of a role of the experimental diet in better preserving the animals’ health. In this context, however, the study conducted by Boato et al. (2002) [27] must be taken into account, in which the ability of red grape juice to inhibit iron availability was observed, exploiting an in vitro digestion/Caco-2 cell model. This finding suggest the presence of anti-nutritional compounds in grape.

The sequencing and characterization of the whole blood transcriptome highlighted the presence of five DEGs between the two experimental groups. Notably, GP dietary supplementation was shown to be able to down-regulate the expression of PLXNC1, ETNK1, TAX1BP1, TM9SF2, and BECN1. The analysis of the interaction network with STRING software did not highlight the ability of these proteins to directly interact in the same metabolic pathways; however, the individual observed interactions allowed interrogation of what has been already reported in the literature, which testifies to the general involvement of these factors in mechanisms of an oxidative and inflammatory nature.

In detail, PLXNC1 encodes a member of the plexin family, a group of transmembrane receptors for semaphorins, which hat regulate cell motility and migration, axon guidance, and the immune response. In a mouse model of Zymosan A (ZyA)-induced peritonitis, it was demonstrated that the plexin C1 receptor promotes acute inflammation [28]. ETNK1 encodes for an ethanolamine kinase, a cytosolic enzyme specific for ethanolamine and with a negligible kinase activity on choline, and directly involved in the first committed step of the phosphatidylethanolamine synthesis pathway. In CLFP mice, a potential role of dietary phosphatidylethanolamine and phosphatidylcholine in favoring the remission of acute inflammation has been demonstrated [29]. TAX1BP1 encodes a HTLV-1 tax1-binding protein that is involved in the inhibition of TNF-induced apoptosis; furthermore, this factor may also have a role in the inhibition of inflammatory events, especially because of its ability to influence the NFĸB pathway [30]. TM9SF2 encodes a 76 kDa member of the transmembrane 9 superfamily, which may play a role in small molecule transport or act as an ion channel. The over-expression of factors belonging to this family of proteins seems to be responsible for inflammation and oncogenic activity [31]. Finally, BCN1 encodes a protein, Beclin-1, that is part of the phosphatidylinositol-3-kinase (PI3K) complex involved in vesicle-trafficking processes and in the regulation of autophagy, a catabolic process of degradation induced by starvation. This protein is thought to play a role in multiple cellular processes, including tumorigenesis, neurodegeneration, and apoptosis. Specific studies demonstrated a reduction in the inflammation level by suppressing the ERK/Beclin-1 signaling [32].

On the basis of what has been described, it appears quite interesting that all these factors have in common the participation in biochemical cascades that are mostly functional in the triggering and progression of inflammatory events. Therefore, it may be reasonable to think that the down-regulation of these factors represents a useful aspect to support an improvement in the general physiological conditions of the animals that received the dietary supplementation. Starting from this premise, it was considered useful to characterize some markers of inflammation and of the antioxidant response at the blood serum level. Specifically, attention was focused on elements that are constitutively expressed and, therefore, are active even in physiological conditions, and not necessarily in the presence of a response to a harmful phenomenon.

The activity of GPx and CAT, as well as the accumulation of IL-1 and TNF-α, were found to not be influenced by the dietary supplementation; however, the findings of the zymographic analysis were interesting, which aimed at defining the gelatinase activity attributable to MMP-2 (gelatinase-A) and MMP-9 (gelatinase-B), two zinc-dependent endopeptidases physiologically involved in tissue remodeling, and which are overexpressed in the presence of tissue damage, inflammation, and neoplastic invasion [33]. Following GP intake by lactating ewes, no significant differences were found for the MMP-2 activity, while a marked reduction in MMP-9 function was observed. Such behavior was consistent with that already observed in various inflammatory mechanisms, as well as in tumor invasion and migration, in which MMP-9 showed much more marked variations, even in the presence of mild stimuli [34]. Since the investigation performed on the transcripts did not highlight the differential expression of the gelatinases or factors strictly related to their activation, it is plausible that the reduction in activity observed for MMP-9 is attributable to the presence in the blood of compounds originating from the diet capable of interacting with the enzyme, influencing its interaction with the substrate and therefore its kinetics. In support of what has been stated, it should be noted that we recently published a work consistent with this line of research, in which ultra-high-performance liquid chromatography analysis highlighted the presence of significantly higher concentrations of luteolin in the milk of the experimental group [35]. This compound, following in silico evaluations, appeared to be able to interact with the active site of MMP-9, allowing us to hypothesize that there was a plausible reduction in the enzyme affinity for the substrate, therefore negatively regulating its function. In addition to this, there are a whole series of studies focused on the modulatory properties of bioactive elements obtained from GP with respect to metalloproteinase function. A relevant example from this point of view is represented by grape-seed-derived procyanidin, which was found to be able to reduce MMP-9 activity in adult male mice with cisplatin-induced blood–brain barrier damage [36]. Furthermore, MMP-9 activity also reduced in glioblastoma cells (GBM 8901) after treatment with different concentrations of naringenin, an ingredient of citrus that is also well represented in grapes [37]. In addition to these examples are studied attributes that highlighted the ability of grape flavonoids to limit the gene expression of MMP-9. Specifically, reference is made to experiments focused on, above all, the role of resveratrol, which is one of the most represented phenolic compounds in red grapes [38].

In conclusion, this study presents the whole blood transcriptome sequencing of ewes fed a 10% GP supplementation. The findings suggest that the experimental diet is responsible for variations in the expression of genes involved, with different magnitudes, in the inflammation pathway. In addition, this study provides evidence of a reduction in serum MMP-9 activity, a constitutively expressed factor involved in both physiological and pathological functions. From this point of view, the aspects regarding the effect of GP-derived phenolic compounds in reducing MMP-9 gene expression, or a synergy between this event and the direct inhibition of enzyme activity, deserve to be better and specifically characterized.

## Figures and Tables

**Table 1:** Complete whole blood count evaluated in lactating ewes fed a standard diet (CG; n = 5) and ewes that received the grape pomace dietary supplementation (EG; n = 5).

	CG	EG	SEM	*p*
WBC, ×10^3^ µL^−1^	9.88	8.60	0.95	0.15
RBC, ×10^6^ µL^−1^	10.18	9.83	0.55	0.67
HGB, g dL^−1^	11.48	11.88	0.48	0.62
HCT, %	27.67	27.77	1.33	0.95
MCV, fl	27.25	28.62	1.28	0.37
MCH, pg	11.30	12.13	0.54	0.11
MCHC, g dL^−1^	41.52	42.63	1.18	0.42
RDW, %	18.28	18.50	0.45	0.76
HDW, g dL^−1^	2.97	2.98	0.15	0.99
PLT, ×10^3^ µL^−1^	989.00	1169.17	161.01	0.34
MPV, fl	17.20	16.93	1.31	0.85
Neutrophils, %	39.62	35.48	4.45	0.29
Lymphocytes, %	45.35	47.52	5.32	0.63
Monocytes, %	4.75	6.45	1.18	0.12
Eosinophils, %	7.30	7.77	1.71	0.77
Basophils, %	2.03	2.13	0.20	0.66
LUC, %	0.90	0.62	0.21	0.14

Data are reported as mean ± pooled standard error (SEM). WBC: white blood cell count; RBC: red blood cell count; HGB: hemoglobin; HCT: hematocrit, MCV: mean corpuscular volume; MCH: mean corpuscular content hemoglobin; MCHC: mean corpuscular hemoglobin concentration, RDW: volume distribution index RBC; HDW: concentration distribution index HGB; PLT: platelet count; MPV: mean platelet volume; LUC: large uncolored cells.

**Table 2:** Blood serum biochemistry of lactating ewes fed a standard diet (CG; n = 5) and ewes that received the grape pomace dietary supplementation (EG; n = 5).

	CG	EG	SEM	*p*
GOT (AST), IU L^−1^	147.83	143.00	42.36	0.49
Triglycerides, mg dL^−1^	17.17	16.83	3.30	0.89
Proteins, g dL^−1^	7.52	7.28	0.97	0.21
Amylase, IU L^−1^	37.67	35.83	7.71	0.90
γ GT, IU L^−1^	59.40	79.23	20.62	0.55
GPT (ALT), IU L^−1^	20.67	18.00	3.65	0.25
Albumin, g dL^−1^	3.57	3.63	0.69	0.24
Azotemia, mg dL^−1^	22.50	22.83	4.64	0.89
Cholesterol, mg dL^−1^	65.17	70.33	6.22	0.37
Glucose, mg dL^−1^	64.83	61.67	3.58	0.42
Bilirubin, mg dL^−1^	0.02	0.08	0.50	0.09
Creatinine, mg dL^−1^	0.97	0.95	0.55	0.77
Alkaline Phosphatase, IU L^−1^	119.17	120.17	13.89	0.98
Calcium, mg dL^−1^	10.32	10.32	1.14	1.00
Uric Acid, mg dL^−1^	0.12	0.12	0.33	1.00
Iron, µg dl^−1^	130.00	166.17	13.13	0.07

Data are reported as mean ± pooled standard error (SEM).

**Table 3:** Differentially expressed genes (DEGs) in EG samples compared with CG samples by combining a false discovery rate (FDR) lower than 0.05 and a log_2_ fold change (log_2_FC) higher than 0.5 or lower than −0.5.

Reference Sequence	BaseMean	log_2_FoldChange	FC	FoldChange	lfcSE	*p*-Value	FDR
exon-XM_015094600.2-29	16.25	−0.37	0.78	−1.29	1.12	1.58 × 10^−14^	8.67 × 10^−11^
exon-XM_012175197.2-6	14.99	−0.37	0.77	−1.29	1.12	1.58 × 10^−14^	8.67 × 10^−11^
exon-XM_012176896.3-8	16.53	−0.36	0.78	−1.28	1.12	2.43 × 10^−14^	8.67 × 10^−11^
exon-XM_004012221.4-10	13.36	−0.37	0.78	−1.29	1.12	2.89 × 10^−14^	8.67 × 10^−11^
exon-XM_004012945.4-8	12.95	−0.37	0.78	−1.29	1.12	3.15 × 10^−14^	8.67 × 10^−11^

BaseMean: mean value of the normalized expression value in sample and control; log_2_FoldChange: ratio of the normalized expression value in sample over control expressed as log_2_; FC: ratio of the normalized expression value in sample over control expressed as real number; FoldChange: ratio of the normalized expression value in sample over control, where values < 1 are shown as (−1/FC) to display the negative behavior of the gene; IfcSE: standard error of ratio of the normalized expression value in sample over control; FDR: false discovery rate.

## Data Availability

Neither the data or models have been deposited in an official repository. The data that support the study findings are available on request.
